# Alanine–Glyoxylate Aminotransferase Sustains Cancer Stemness Properties through the Upregulation of SOX2 and OCT4 in Hepatocellular Carcinoma Cells

**DOI:** 10.3390/biom12050668

**Published:** 2022-05-05

**Authors:** Peng Ye, Xiaoxia Chi, Xiuwen Yan, Fangqin Wu, Zhigang Liang, Wen-Hao Yang

**Affiliations:** 1 Key Laboratory of Cell Homeostasis and Cancer Research of Guangdong Higher Education Institutes and Affiliated Cancer Hospital & Institute, Guangzhou Medical University, Guangzhou 910095, China; 2019217972@stu.gzhmu.edu.cn (P.Y.); xiaoxia@stu.gzhmu.edu.cn (X.C.); sure83@gzhmu.edu.cn (X.Y.); 2019217958@stu.gzhmu.edu.cn (Z.L.); 2 Key Laboratory for Reproductive Medicine of Guangdong Province, The Third Affiliated Hospital of Guangzhou Medical University, Guangzhou 910095, China; 2019217679@stu.gzhmu.edu.cn; 3 Graduate Institute of Biomedical Sciences, China Medical University, Taichung 406040, Taiwan

**Keywords:** hepatocellular carcinoma, cancer stem cells, AGXT, SOX2, OCT4

## Abstract

Liver cancer stem cells (LCSCs) are a small subset of oncogenic cells with a self-renewal ability and drug resistance, and they promote the recurrence and metastasis of hepatocellular carcinoma (HCC). However, the mechanisms regulating LCSCs have not been fully explored. By enriching LCSCs from spheroid cultures and performing transcriptomic analysis, we determined that alanine–glyoxylate aminotransferase (AGXT), which participates in the metabolism of serine and glycine, was significantly upregulated in spheroid cultures, and its function in LCSCs remains unknown. Through the exogenous overexpression or short hairpin RNA knockdown of AGXT in HCC cells, we observed that changes in the AGXT level did not affect the spheroid ability and population of LCSCs. The knockdown of AGXT in LCSCs reduced the number of spheroids and the population of LCSCs; this implies that AGXT is required for the maintenance of cancer stemness rather than as a driver of LCSCs. Mechanistically, AGXT may sustain the self-renewal potential of LCSCs by upregulating the expression of SRY-box transcription factor 2 (SOX2) and octamer-binding transcription factor 4 (OCT4), two well-known master regulators of cancer stemness. Taken together, our study demonstrates the role of AGXT in supporting LCSCs; thus, AGXT merits further exploration.

## 1. Introduction

Primary liver cancer is among the most common cancers globally; its mortality rate ranks third among all malignant tumors [1], and hepatocellular carcinoma (HCC) is the most crucial type of primary liver cancer. The treatment options for HCC are diverse, including surgical resection and transplantation, radiation therapy, chemotherapy, transcatheter arterial chemoembolization, tyrosine kinase inhibitor use, and immunotherapy [2]. However, despite the availability of complex and comprehensive treatment strategies that improve overall survival, HCC is often difficult to cure and prone to treatment resistance and recurrence [3].

Cancer stem cell (CSC) theory provides new insights for treating tumors. CSCs often possess drug resistance and self-renewal properties, and are considered to be the root cause of tumor recurrence. Targeting CSCs may be an effective strategy to overcome the barriers to curing HCC [4]. CSCs are less abundant in tumors, and can be enriched in vitro by performing spheroid formation assays by using serum-free stem cell medium. This population of spheroid cells would possess the properties of CSCs, and this method has been used in HCC [5,6], glioblastoma [7], ovarian cancer [8] and breast cancer [9]. In addition, CSCs can be identified and isolated by using biomarkers. CSC biomarkers that have been identified in HCC, including CD133, CD90, CD44, Nanog, octamer-binding transcription factor 4 (OCT4), and SRY-box transcription factor 2 (SOX2), have been reported to contribute to the maintenance of stemness [10]. CSC biomarkers can be targeted to eliminate CSCs [11]. However, currently, eliminating CSCs is challenging. One of the difficulties is that the regulatory mechanism of CSCs is complex and not fully understood. Therefore, the identification of genes involved in their self-renewal properties can provide insights into the blockade of HCC progression by targeting cancer stemness.

Alanine–glyoxylate and serine–pyruvate aminotransferase (AGXT) is the gene encoding alanine–glyoxylate aminotransferase (AGT). AGT, localized in hepatic peroxisomes, is involved in glyoxylate detoxification, and its physiological role is to catalyze the conversion of L-alanine and glyoxylate to pyruvate and glycine, respectively. Mutations in the AGXT cause glyoxylate metabolism disorders and excessive oxalate accumulation, eventually leading to primary hyperoxaluria type I [12]. AGXT rs34116584 was reported to affect the hepatic peroxisomal metabolism of platinum-based chemotherapeutics, and is associated with poor prognosis in metastatic colorectal cancer [13] and non-small-cell lung cancer [14]. Amino acid metabolic reprogramming is crucial for maintaining the self-renewal and drug-resistance properties of CSCs [15]. Another study reported that a decreased alanine level and increased serine and glycine levels promoted the oncogenic potential of nonadherent tumor spheroids in vitro and in vivo [16]. In this study, we observed that AGXT was significantly upregulated in spheroid cultures. AGXT may maintain the self-renewal capacity of liver CSCs (LCSCs) by upregulating the stemness transcription factors SOX2 and OCT4. Given that AGXT is involved in the metabolism of serine and glycine, our findings may indicate an association of amino acid metabolism with LCSCs.

## 2. Materials and Methods

### 2.1. Cell Line Culture

All cell lines were purchased from the cell bank of Shanghai Institute for Biological Science, Chinese Academy of Sciences (Shanghai, China); they were authenticated through short-tandem-repeat profiling and regularly checked for mycoplasma contamination. HEK-293T cells and human hepatocellular carcinoma Huh7, HepG2, Hep3B, and MHCC-97H cells were cultured in Dulbecco’s modified Eagle’s medium (DMEM; BI, Kibbutz Beit HaEmek, Israel). Human hepatocellular carcinoma Bel7402 and SMMC7721 cells were cultured in RPMI 1640 medium (Gibco, Carlsbad, CA, USA). Human hepatocellular carcinoma PLC/PRF/5 cells were cultured in minimum essential medium (Gibco, Carlsbad, CA, USA). All the media were supplemented with 10% inactivated fetal bovine serum (04-001-1ACS; BI) and 1% penicillin–streptomycin (Cat. 15140122; Thermo Fisher Scientific Inc., Waltham, MA, USA), and all the cells were incubated in a 37 °C incubator under 5% carbon dioxide.

### 2.2. RNA Sequencing

In this study, RNA sequencing (RNA-seq) was performed with Novogene (Beijing, China) using the Illumina Hiseq XTEN platform. RNA-seq data were deposited in the GEO (www.ncbi.nlm.nih.gov/geo/, accessed on 1 July 2022) with the GEO series number GSE199940, and can be accessed by entering secure token utoleaeghvifxct into the box.

### 2.3. Data Source and Software

The data of patients with HCC were obtained from the Cancer Genome Atlas Liver HCC cohort (TCGA-LIHC; https://tcga-data.nci.nih.gov/tcga/, accessed on 11 March 2021). AGXT expression in the TCGA-LIHC cohort was divided into AGXT-High and AGXT-Low expression groups based on median values. Gene set enrichment analysis (GSEA) was performed using GSEA software (version 4.1.0; http://software.broadinstitute.org/gsea, accessed on 2 August 2020). C2 (c2. cp. kegg. V 7.5. symbols. gmt) from the Molecular Signatures Database (MSigDB) was used as the reference gene set. The number of permutations was set to 1000.

### 2.4. Quantitative Real-Time Polymerase Chain Reaction

Total RNA was extracted from the cells using the total RNA isolation kit (RC101-01, Vazyme, Nanjing, China) according to the manufacturer’s instructions. Complementary DNA (cDNA) was generated using the RevertAid Master Mix reagent (M1631, Thermo Fisher Scientific Inc., Waltham, MA, USA). Real-time quantitative polymerase chain reaction (PCR) was performed on the CFX96 Real-Time PCR System (Bio-rad, Hercules, CA, USA) using TB Green Premix Ex Taq II (Cat. RR820A, TaKaRa, Shiga, Japan). Details regarding all the gene primers used in this study are available in Supplementary Materials (Table S1).

### 2.5. Sphere Formation Assay

The sphere formation assay was performed using a previously described method [17]. For spherical culture, DMEM/F12 medium supplemented with 2% B27 (Gibco, Carlsbad, CA, USA), 20 ng/mL epidermal growth factor (Peprotech, Rocky Hill, NJ, USA), and 20 ng/mL basic fibroblast growth factor (Peprotech, Rocky Hill, NJ, USA) was used as the serum-free medium.

### 2.6. Western Blot

The harvested cells were lysed in RIPA lysis buffer (Cat. C500005, Sangon Biotech, Shanghai, China) at 4 °C to obtain protein samples, and protein concentrations were determined using the Pierce bicinchoninic acid protein assay kit (Cat. 2322, Thermo Fisher Scientific Inc., Waltham, MA, USA). The protein samples were electrophoretically separated through sodium dodecyl sulfate–polyacrylamide gel electrophoresis, transferred onto polyvinylidene fluoride membranes, and blocked with 5% nonfat milk in TBST. The membranes were incubated with primary antibodies overnight at 4 °C, followed by horseradish peroxidase-labeled secondary antibodies. Antibody binding was visualized using Western blotting luminol reagent (sc-2048, Santa Cruz, Biotechnology, Santa Cruz, CA, USA) on a Tanon-5200 chemiluminescent imaging system (Tanon, Shanghai, China). The following antibodies were used in this study: AGXT (Cat. ab261910, 1:1000, Abcam, Cambridge, MA, USA), OCT4 (Cat. GTX101497, 1:5000, GeneTex, Irvine, CA, USA), Nanog (Cat.GTX627421, 1:1000, GeneTex, Irvine, CA, USA), SOX2 (Cat. GTX101507, 1:5000, GeneTex, Irvine, CA, USA), CD133 (Cat. ab19898, 1:1000, Abcam, Cambridge, MA, USA), β-tubulin (Cat.HRP-66240, 1:20000, Proteintech, Chicago, IL, USA), β-actin (Cat.HRP-60008, 1: 5000, Proteintech, Chicago, IL, USA), and goat antirabbit (Cat. 7074S, 1:3000, Cell Signaling Technology, Inc., Beverly, MA, USA).

### 2.7. Flow Cytometry Analysis and Cell Sorting

The cells were analyzed using a FACSVerse flow cytometer (BD Biosciences, San Jose, CA, USA) and sorted with a FACSAria II cell sorter (BD Biosciences, San Jose, CA, USA). Antibodies used for the flow analysis or sorting were as follows: anti-CD133 (PE, Cat. 566593, BD Pharmingen, San Jose, CA, USA) and anti-CD90 (APC, Cat.559869, BD Pharmingen, San Jose, CA, USA). Data analysis was performed using FlowJo software (Version 10, LLC, Ashland, OR, USA).

### 2.8. Animal Study

NOD/SCID male mice (5-week-old) were purchased from GemPharmatech Co., Ltd. (Jiangsu, China). These mice were housed under pathogen-free conditions. All mouse experiments were performed in accordance with animal welfare guidelines and approved by the Institutional Animal Care and Use Committee of Guangzhou Medical University.

### 2.9. Statistical Analysis

All statistical data were derived from at least three independent experiments to avoid experimental errors. Data are presented as mean ± standard deviation, and significant differences were analyzed using a two-tailed independent Student’s *t* test. A *p* value of < 0.05 was considered statistically significant.

## 3. Results

### 3.1. Identification of Differentially Expressed Genes between Spheroid Culture Cells and Adherent Cells

**Figure 1:**
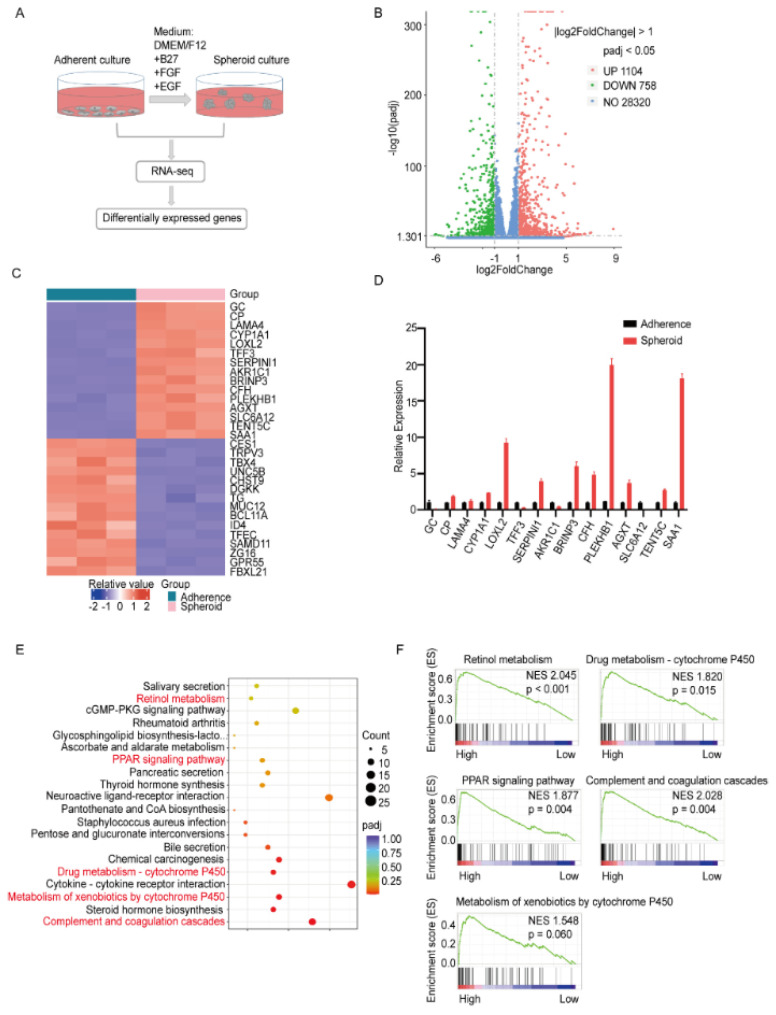
Identification of differentially expressed genes (DEGs) between spheroid and adherent cells: (**A**) A schematic outline for the identification of differential genes in spheroid and parental cells. Huh7 cells were plated on a serum-free medium in ultra-low-attachment dishes (Corning). The spheroids and adherent parental cells were harvested and subjected to transcriptome sequencing; (**B**) a volcano plot showing genes differentially expressed in the adherent and spheroid cell groups. The red dots represent upregulated genes (1104), the green dots represent downregulated genes (756), and the blue dots represent non-DEGs (28320); (**C**) a heatmap showing the top 15 genes with the most upregulation and downregulation; (**D**) quantitative real-time polymerase chain reaction of the top 15 genes in the adherent cells and spheroid cells; (**E**) bubble plot of the Kyoto Encyclopedia of Genes and Genomes (KEGG) enrichment analysis of upregulated DEGs. The ordinate represents the top-20 upregulated pathways, the color of the bubble represents the size of the *p* value, and the size of the bubble represents the number of enriched differential genes; (**F**) gene set enrichment analysis of KEGG signaling pathways in the high alanine−glyoxylate aminotransferase expression group of the Cancer Genome Atlas–Liver Hepatocellular Carcinoma cohort.

We first enriched LCSCs by culturing the Huh7 cells in ultra-low-attachment surface plates with a serum-free medium to form spheroid culture cells. To identify the relevant genes regulating LCSCs, RNA-seq was performed using the spheroid and adherent culture cells (Figure 1A). By comparing transcriptome sequences, we identified 1860 differentially expressed genes in the spheroid culture cells compared with the adherent culture cells. Volcano plots indicated the presence of 1104 upregulated genes and 756 downregulated genes in the spheroid culture cells compared with the adherent culture cells (Figure 1B). The top 15 upregulated genes and the top 15 downregulated genes are displayed using a heatmap (Figure 1C). Furthermore, we validated the expression of the top 15 upregulated genes through quantitative real-time PCR (qRT-PCR) in the spheroid cells compared with the adherent cells. We observed that 10 genes were upregulated in the spheroid cells; this finding is consistent with that of RNA-Seq (Figure 1D). Among these genes, AGXT was related to amino acid metabolism. To the best of our knowledge, no study has reported the association of AGXT with CSC regulation. Moreover, the findings of the Kyoto Encyclopedia of Genes and Genomes (KEGG) pathway enrichment analysis, performed using the genes upregulated in the RNA-Seq data, revealed that among the top 20 enriched pathways, some have been reported to be involved in stemness regulation, including retinol metabolism [18], peroxisome proliferator-activated receptor signaling [7], cytochrome P450 metabolism [8], and complement and coagulation cascades [19] (Figure 1E). Next, we downloaded the TCGA-LIHC cohort data and conducted gene set enrichment analysis (GSEA) on the basis of the expression level of AGXT. The results indicated that the aforementioned five pathways were all enriched in the AGXT-High expression group (Figure 1F). Collectively, these data indicate that AGXT may play a crucial role in regulating the biological function of LCSCs in spheroid cultures.

### 3.2. AGXT Expression Cannot Drive Cancer Stemness in HCC Cells

**Figure 2:**
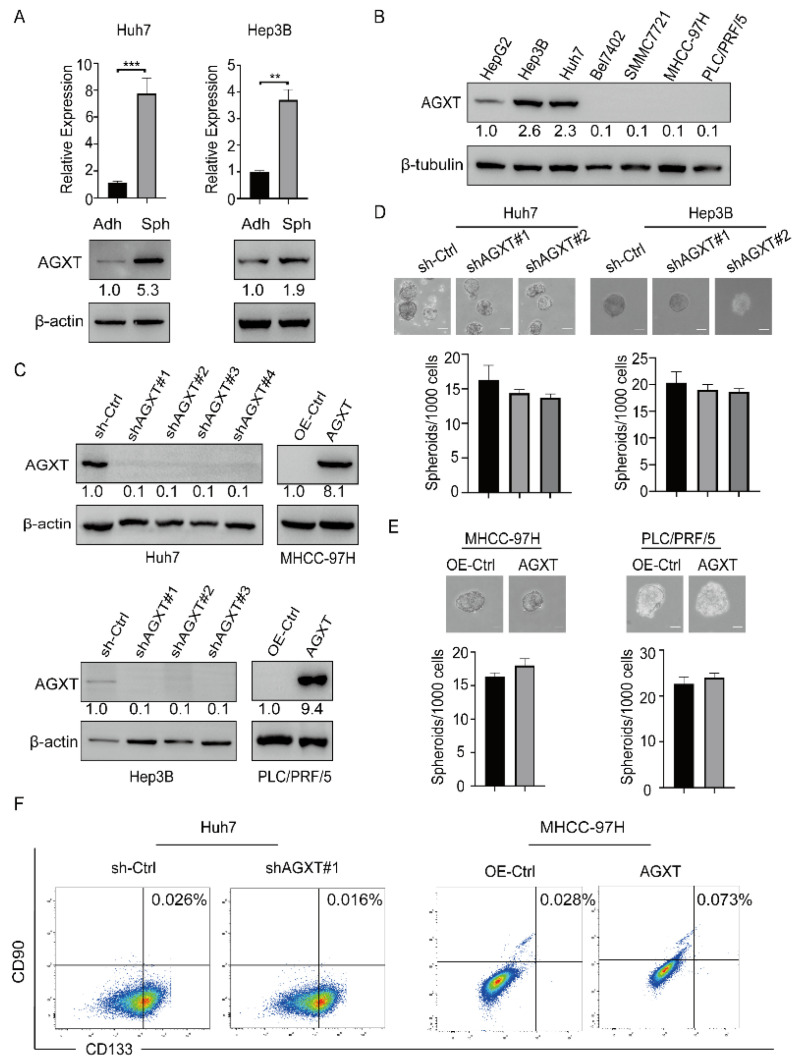
Alanine−glyoxylate aminotransferase (AGXT) expression cannot drive the stemness of hepatocellular carcinoma (HCC) cells: (**A**) Quantitative real−time polymerase chain reaction and Western blot analysis of spheroid cells (Sph) and adherent cells (Adh) among the Huh7 and Hep3B cells; (**B**) Western blot analysis of AGXT in seven HCC cell lines (HepG2, Hep3B, Huh7, Bel7402, SMMC7721, MHCC−97H, and PLC/PRF/5); (**C**) Western blot exhibited the knockdown of AGXT in the Huh7 cells and Hep3B cells and the exogenous overexpression of AGXT in the MHCC−97H cells and PLC/PRF/5 cells; (**D**,**E**) sphere formation assay of stable AGXT knockdown cell lines and AGXT-overexpressing cell lines (top panel). Statistical bar graphs of sphere numbers (down panel). Scale bar, 100 μm; (**F**) flow cytometric analysis of membrane CD133^+^/CD90^+^ in Huh7 cells and MHCC−97H cells. ** *p* < 0.01, *** *p* < 0.001.

To determine whether AGXT expression affects HCC stemness, we first examined the expression of AGXT by performing the anchorage-independent spheroid formation assay in two HCC cell lines, Huh7 and Hep3B. The results of both qRT-PCR and Western blotting revealed that AGXT expression increased in the Huh7 and Hep3B spheroid cells (Sph) compared with their adherent (Adh) cells (Figure 2A). To select suitable cell lines for genetic manipulation, we determined the protein expression of AGXT in several HCC cell lines through Western blotting. The findings indicated that AGXT was highly expressed in three HCC cell lines, namely Huh7, HepG2, and Hep3B (Figure 2B). To investigate whether altered AGXT expression affects the stemness characteristics of the HCC cells, we knocked down AGXT expression by using short hairpin RNA (shRNA) in the Huh7 and Hep3B cells, and exogenously upregulated AGXT expression in the MHCC-97H and PLC/PRF/5 cells (Figure 2C). Furthermore, we performed the sphere formation assay by using these cell lines with altered AGXT expression. The sphere-forming efficiency (SFE) of the spheroid culture in the AGXT-knockdown Huh7 and Hep3B cells (sh-AGXT) exhibited no significant changes compared with the control cells (sh-Ctrl; Figure 2D). Consistently, AGXT overexpression in the MHCC-97H and PLC/PRF/5 cells did not affect the SFE of the spheroid culture compared with the control cells (OE-Ctrl; Figure 2E). In addition, we performed flow cytometry to investigate the effect of AGXT expression on the CD113^+^CD90^+^ cell population, a specific cell population of LCSCs [20]. The data indicated that neither the knockdown of AGXT in the Huh7 cells nor the overexpression of AGXT in the MHCC-97H cells significantly affected the number of CD113^+^CD90^+^ cells (Figure 2F). Furthermore, we determined the effect of AGXT on tumor migration, colony formation in soft agar, and drug resistance. The results of the wound-healing assay and transwell migration assay indicated that AGXT overexpression in the Huh7 or MHCC-97H cells did not affect cell migration ability (Supplementary Figure S1A–C). Moreover, AGXT expression in the Huh7 cells did not alter the number of colonies formed on soft agar, and it was not involved in drug resistance (Supplementary Figure S1D,E). Taken together, these data suggest that AGXT does not directly increase the capacity of cancer stemness to promote HCC malignancy.

### 3.3. AGXT Expression Is Essential for Sustaining the Stemness Properties of LCSCs

**Figure 3:**
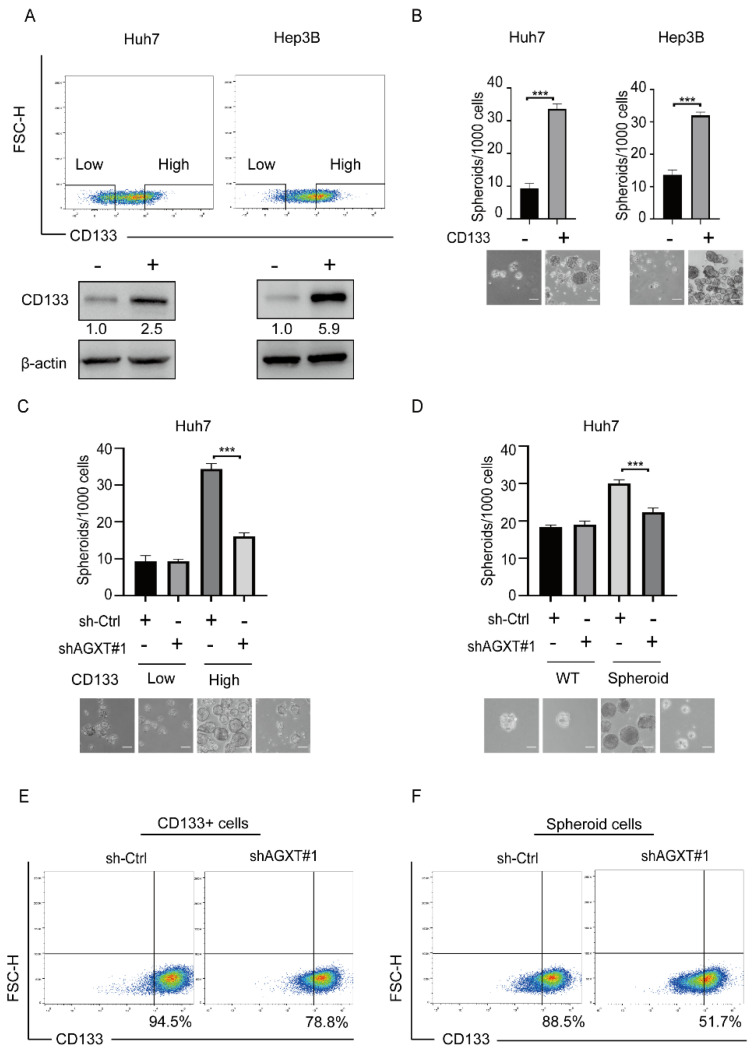
Alanine−glyoxylate aminotransferase (AGXT) expression is essential for the stemness properties of cancer stem cells: (**A**) Differentiation of CD133^+^ cells and CD133^−^ cells through flow cytometry in the Huh7 cells and Hep3B cells. The protein level of CD133 was detected through Western blot after the culture; (**B**) sphere formation assay of CD133^+^ and CD133^−^ Huh7 and Hep3B cells and the quantification of spheroid numbers. Scale bar, 100 μm; (**C**) sphere formation assay of AGXT knockdown and knockdown control in CD133^+^ and CD133^−^ Huh7 cells and the quantification of spheroid numbers. Scale bar, 100 μm; (**D**) sphere formation assay of AGXT knockdown in wild−type and spheroid Huh7 cells, and the quantification of spheroid numbers. Scale bar, 100 μm; (**E**) flow cytometry analysis of the CD133^+^ population decreased from 94.5% to 78.8% after AGXT knockdown; (**F**) flow cytometry analysis of spheroid cells decreased from 86.5% to 51.7% after AGXT knockdown. *** *p* < 0.001.

Because AGXT expression cannot drive cancer stemness, we speculated that AGXT is involved in the maintenance of LCSCs. To test this possibility, we enriched LCSCs and examined whether altered AGXT expression affects their self-renewal capacity. A previous study reported that CD133^+^ cells (high CD133 expression) possess the properties of LCSCs, and that CD133^−^ cells (low CD133 expression) are non-LCSCs [21]. We used the flow cytometry cell-sorting strategy to isolate viable CD133^+^ cells from CD133^−^ cells among the Huh7 and Hep3B cells (Figure 3A). We confirmed that the CD133^+^ cells had a higher SFE than did the CD133^−^ cells, implying that the CD133^+^ cells had stronger stem-like features (Figure 3B). The knockdown of AGXT in the CD133^+^ Huh7 cells reduced the SFE; however, the knockdown of AGXT in the CD133^−^ Huh7 cells did not affect the SFE (Figure 3C). Because the anchorage-independent spheroid cells of HCC have been reported to possess stemness properties and are considered beneficial to the enrichment of LCSCs [5,6], we knocked down AGXT in the Huh7 spheroid cells, then performed a spheroid formation assay. Compared with AGXT knockdown in the wild-type Huh7 cells, AGXT knockdown in the Huh7 spheroid cells significantly reduced the SFE (Figure 3D). Moreover, the findings of flow cytometry indicated that the knockdown of AGXT in the CD133^+^ Huh7 cells or spheroid cells substantially reduced the number of CD133^+^ cells (Figure 3E,F). Together, these results imply that AGXT is involved in maintaining the stemness of LCSCs.

### 3.4. AGXT Upregulates Stemness Transcription Factors to Maintain the Stemness Properties of LCSCs

**Figure 4:**
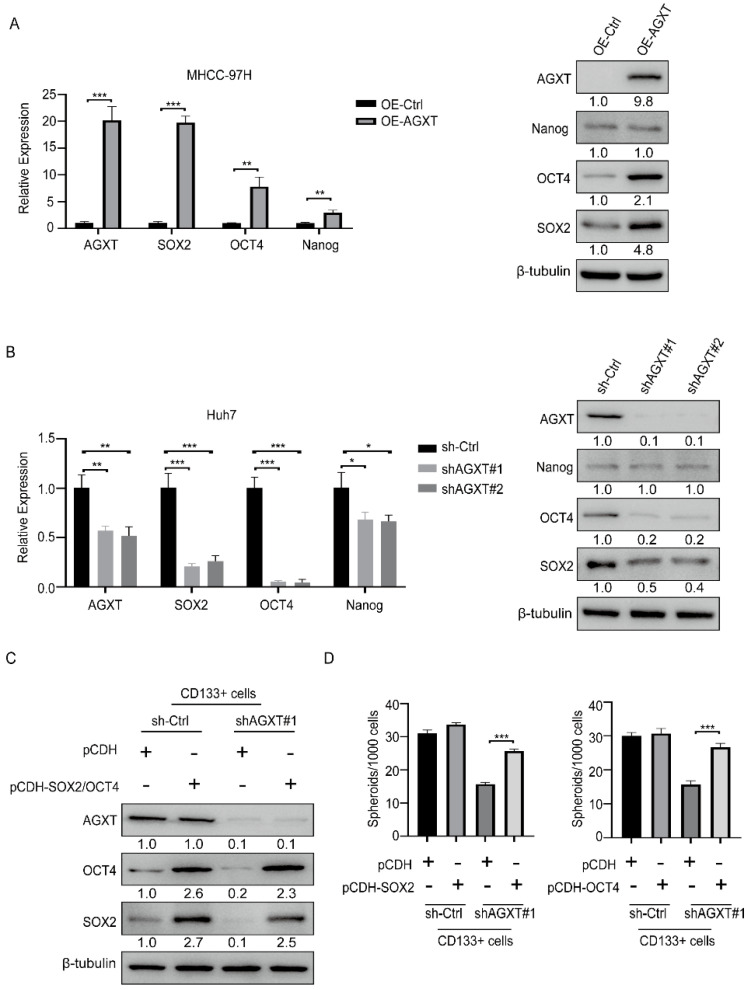
Alanine−glyoxylate aminotransferase (AGXT) upregulates stemness transcription factors to maintain stemness properties in liver cancer stem cells: (**A**) quantitative real−time polymerase chain reaction (qRT−PCR) and Western blot analysis of transcription factors in AGXT−overexpressing MHCC97 cells; (**B**) qRT−PCR and Western blot analysis of the expression of transcription factors in AGXT knockdown Huh7 cells; (**C**) Western blot analysis of SRY−box transcription factor 2 (SOX2) and octamer−binding transcription factor 4 (OCT4) overexpression, and AGXT knockdown, in CD133^+^ cells; (**D**) statistical bar graphs of sphere numbers in SOX2-overexpressing or AGXT knockdown CD133^+^ cells (left) and OCT4−overexpressing or AGXT knockdown CD133^+^ cells. Scale bar, 100 μm. * *p* < 0.05, ** *p* < 0.01, *** *p* < 0.001.

Because a previous study reported that three crucial pathways (Hedgehog, Notch, and Wnt) regulate CSC activity [22], we investigated whether AGXT expression is involved in the regulation of these pathways. The results of qRT-PCR indicated that AGXT overexpression in the Huh7 cells did not alter the mRNA expression of the critical genes of the Hedgehog, Notch, and Wnt pathways, implying that AGXT does not regulate these pathways (Supplementary Figure S1F). Because the stemness-related transcription factors SOX2, OCT4, and Nanog have been demonstrated to sustain the stemness state of CSCs [20,23], we explored whether AGXT affects the mRNA levels of these stemness-related transcription factors. We observed that AGXT overexpression in the MHCC-97H cells upregulated the mRNA and protein levels of SOX2 and OCT4 (Figure 4A). Consistently, the knockdown of AGXT in the Huh7 cells reduced the mRNA and protein levels of these two genes (Figure 4B). Therefore, we speculate that SOX2 and OCT4 mediate the maintenance of LCSC properties through AGXT. To test this hypothesis, we exogenously re-expressed SOX2 or OCT4 in the AGXT-knockdown or control CD133^+^ Huh7 cells (Figure 4C), then used these cells in the spheroid formation assay. The SFE reduced by AGXT knockdown in the CD133^+^ Huh7 cells, compared with the control cells, was partially restored through the re-expression of SOX2 or OCT4 (Figure 4D). These data suggest that AGXT upregulates the expression of SOX2 and OCT4 to support the stemness properties of LCSCs.

### 3.5. Knockdown of AGXT Reduces the Tumorigenic Capability of LCSCs In Vivo

**Figure 5:**
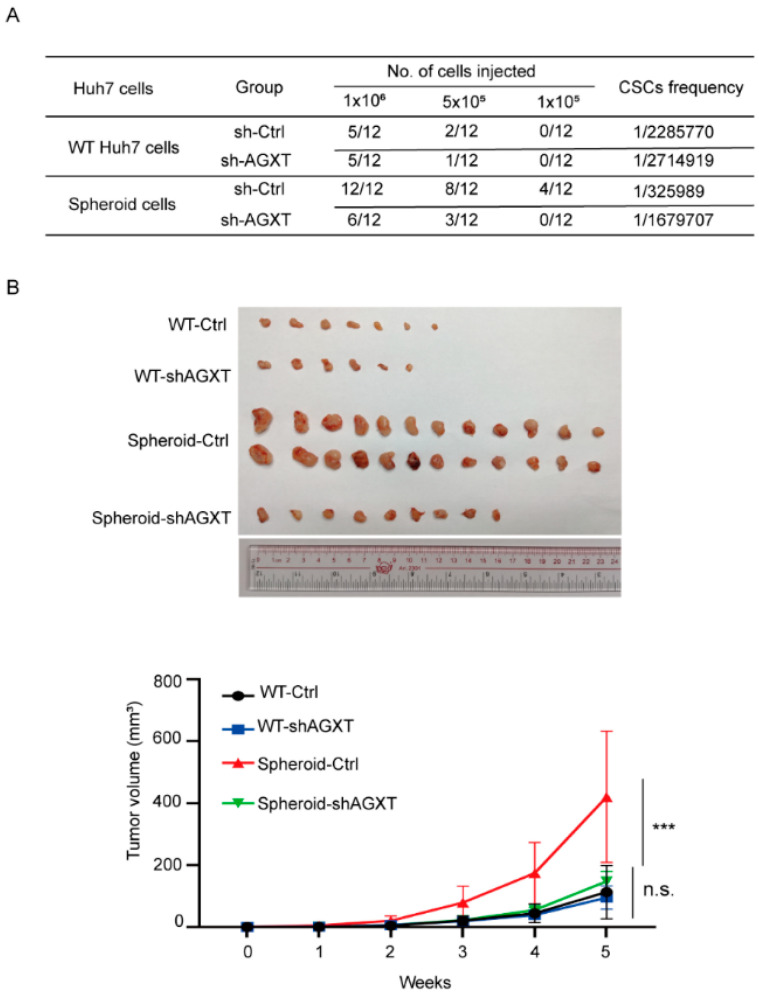
Knockdown of alanine−glyoxylate aminotransferase (AGXT) reduces the oncogenic ability of liver cancer stem cells (CSCs) in mice: (**A**) Adherent and spheroid Huh7 cells with or without AGXT knockdown were subcutaneously injected (10^6^, 5 × 10^5^, and 10^5^ cells per mouse) into 5-week-old male NOD/SCID mice. Tumor formation ability and CSC frequency were analyzed; (**B**) the tumor volume of orthotopic xenograft models at 5 weeks (up panel). Tumor growth curve of adherent and spheroid Huh7 cells with or without AGXT (down panel). *** *p* < 0.001; n.s, nonsignificant.

We performed xenograft experiments to investigate the in vivo role of AGXT in tumorigenesis. We used four inoculated cells to establish the animal model, namely: the control group of parental Huh7 cells (WT-Ctrl), the AGXT knockdown group of parental Huh7 cells (WT-shAGXT), the control group of Huh7 spheroid cells (Spheroid-Ctrl), and AGXT knockdown group of Huh7 spheroid cells (Spheroid-shAXGT). Various numbers of cells were subcutaneously transplanted into the backs of the NOD/SCID male mice. The mice in the Spheroid-Ctrl group still developed tumors, whereas the mice in the other three groups failed to develop tumors after the inoculation of 10^5^ cells. The tumor formation frequency of the Spheroid-Ctrl group was 100%, whereas those of the other groups were not more than 50% after the inoculation of 10^6^ cells (Figure 5A). AGXT knockdown in the Huh7 cells did not affect the tumorigenic rate in the mice. However, AGXT silencing in the Huh7 spheroid cells significantly reduced the tumor formation rate from 100% to 50% after the inoculation of 10^6^ cells (Figure 5B, top panel). In addition, the CSC frequencies of the four groups were determined using the ELDA webtool (http://bioinf.wehi.edu.au/software/elda/, accessed on 1 April 2022). Compared with the spheroid-Ctrl group, the Spheroid-shAXGT group exhibited a decreased CSC frequency (from 1/325989 to 1/1679707; Figure 5A). Moreover, the tumor volume of the Spheroid-Ctrl group was significantly larger than that of the other three groups. In the Huh7 spheroid cells, AGXT knockdown markedly reduced the tumor growth rate. However, AGXT knockdown in the parental Huh7 cells did not significantly affect the tumor growth rate (Figure 5B, lower panel). These in vivo data strongly support the critical role of AGXT in augmenting the tumor formation and growth rates of LCSCs.

## 4. Discussion

HCC is among the cancers with high mortality, mainly because of its resistance to radiotherapy or chemotherapy and its high recurrence and metastasis rates [3]. CSCs are widely believed to be the underlying cause of these clinical observations [10]. Although many therapeutic drugs targeting CSCs have been developed, their clinical therapeutic effect is still limited [11]. Therefore, discovering more potential mechanisms underlying CSC regulation would be beneficial for developing new strategies to target CSCs. This study revealed new regulatory mechanisms of LCSCs. In this study, we utilized an ultra-low-attachment and serum-free culture system to promote CSC-enriched spheroid formation. The RNA-Seq and qRT-PCR showed that 10 genes were significantly up-regulated in spheroid cells (Figure 1D), half of which were reported to be associated with CSC, such as CP [24], CYP1A1 [25], LOXL2 [26], SERPINI1 [27], and CFH [19]. The unreported genes, including BRINP3, PLEKHB1, AGXT, TENT5C, and SAA1, were used to perform GSEA analysis based on the TCGA-LIHC cohort. We found that many CSC-related pathways only appeared in the AGXT high expression group (Figure 1F). Therefore, we selected AGXT as a candidate gene for further study. The data implied that AGXT does not drive HCC stemness but is crucial for maintaining the tumor-initiating and self-renewal properties of LCSCs.

Previous studies have reported that in colon cancer [13] and lung cancer [14], the AGXT rs34116584 variant affects the metabolism of platinum-based chemotherapeutics, leading to poor prognosis. Another study demonstrated that AGXT depletion accelerated HCC progression and predicted poor prognosis [28]. Another study also found that HCC patients with increased AGXT expression had a better prognosis and a lower rate of recurrence [29]. These studies indicate that the depletion or mutation of AGXT results in poor prognosis for cancer. Considering that the AGXT gene is related to amino acid metabolism, it may be involved in the metabolic reprogramming of cancer cells to affect prognosis. As a heterogeneous and plastic cell population among cancer cells, CSCs exhibit distinct metabolic programming [30]. AGXT has been demonstrated to regulate the metabolic processes of serine, glycine, and alanine, and previous studies have indicated that amino acid metabolism can affect the tumorigenesis and stemness of CSCs [16,31]. On the basis of these data, we speculate that the metabolic effects of AGXT differ between differentiated cancer cells and CSCs. In our study, we observed that AGXT knockdown significantly reduced the self-renewal capacity of the CD133^+^ cells or CSC-enriched spheroid culture compared with the CD133^−^ cells. AGXT knockdown in the spheroid cells significantly reduced the xenograft growth rate and tumor-forming ability in vivo. These findings suggest that compared with HCC cells, the growth of LCSCs may be more dependent on AGXT. However, given that LCSCs typically represent only a small fraction of HCC cells, the upregulation of AGXT in LCSCs may be inconsistent with its poor expression in mass tumors. Here, we demonstrated that AGXT expression in WT HCC cells did not affect stemness, and only in LCSC could it promote stemness (Figure 3C–F). On the other hand, previous studies reported that a high expression of AGXT can improve the prognosis of HCC patients, which indicates that AGXT may have other functions, such as promoting tumor immunity or other unfavorable factors to inhibit cancer progress. The tumor suppressor effect of AGXT in HCC seems to contradict the findings of this study that AGXT promotes stemness in LCSCs. However, these apparently contradictory observations can be reconciled, in that AGXT may exert different tumor-regulatory roles in LCSCs and non-LCSCs. Therefore, whether AGXT is a favorable or unfavorable factor cannot be rashly defined, and more clinical sample observations are needed to evaluate it. Moreover, our data indicate that AGXT upregulated the expression of the transcription factors SOX2 and OCT4, which have been reported to maintain the stemness characteristics of CSCs [20,23]. However, the mechanisms through which AGXT regulates the expression of SOX2 and OCT4 remain unclear. Furthermore, the biological effects of AGXT-induced SOX2 and OCT4 between parental live cancer cells and LCSCs may be different. Therefore, more research should be conducted to fully investigate the detailed mechanism of AGXT in liver cancer progression, which can link amino acid metabolism with LCSCs and liver cancer malignancy.

## 5. Conclusions

Our study demonstrated that AGXT, which is known to participate in amino acid metabolism, cannot directly promote HCC stemness, but is an essential gene to sustain the cancer stemness properties of LCSCs. Furthermore, AGXT may upregulate the stemness transcription factors SOX2 and OCT4 to maintain cancer stemness in LCSCs. Our study implies that the metabolic processes of serine, glycine, and alanine regulated by AGXT may be crucial for LCSCs, and can inspire potential strategies to interfere with the process of amino acid metabolism for combating LCSCs in the future. However, it must be considered that AGXT may act as a tumor suppressor in HCC cells, and targeting it may also cause adverse effects.

## Data Availability

The raw RNA sequencing data in this study have been deposited to the GEO (www.ncbi.nlm.nih.gov/geo/) with the accession number GSE199940.

## Supplementary Materials

The following supporting information can be downloaded at: https://www.mdpi.com/article/10.3390/biom12050668/s1, Figure S1: Elevated expression of AGXT cannot affect tumor migration, proliferation, drug resistance and stemness-related pathways; Table S1: The qRT-PCR primers of genes involved in this article.

## References

[1] Sung H., Ferlay J., Siegel R.L., Laversanne M., Soerjomataram I., Jemal A., Bray F. (2021). Global cancer statistics 2020: GLOBOCAN estimates of incidence and mortality worldwide for 36 cancers in 185 countries. CA Cancer J. Clin..

[2] Nault J.C., Cheng A.L., Sangro B., Llovet J.M. (2020). Milestones in the pathogenesis and management of primary liver cancer. J. Hepatol..

[3] Kulik L., El-Serag H.B. (2019). Epidemiology and Management of Hepatocellular Carcinoma. Gastroenterology.

[4] Chen B., Ye P., Chen Y., Liu T., Cha J.H., Yan X., Yang W.H. (2020). Involvement of the Estrogen and Progesterone Axis in Cancer Stemness: Elucidating Molecular Mechanisms and Clinical Significance. Front. Oncol..

[5] Cao L., Zhou Y., Zhai B., Liao J., Xu W., Zhang R., Li J., Zhang Y., Chen L., Qian H. (2011). Sphere-forming cell subpopulations with cancer stem cell properties in human hepatoma cell lines. BMC Gastroenterol..

[6] Raudenska M., Balvan J., Fojtu M., Gumulec J., Masarik M. (2019). Unexpected therapeutic effects of cisplatin. Metallomics.

[7] Ning J.F., Stanciu M., Humphrey M.R., Gorham J., Wakimoto H., Nishihara R., Lees J., Zou L., Martuza R.L., Wakimoto H. (2019). Myc targeted CDK18 promotes ATR and homologous recombination to mediate PARP inhibitor resistance in glioblastoma. Nat. Commun..

[8] Li J., Condello S., Thomes-Pepin J., Ma X., Xia Y., Hurley T.D., Matei D., Cheng J.X. (2017). Lipid Desaturation Is a Metabolic Marker and Therapeutic Target of Ovarian Cancer Stem Cells. Cell Stem Cell.

[9] Shaw F.L., Harrison H., Spence K., Ablett M.P., Simões B.M., Farnie G., Clarke R.B. (2012). A detailed mammosphere assay protocol for the quantification of breast stem cell activity. J. Mammary Gland. Biol. Neoplasia.

[10] Makena M.R., Ranjan A., Thirumala V., Reddy A.P. (2020). Cancer stem cells: Road to therapeutic resistance and strategies to overcome resistance. Biochim. Biophys. Acta-Mol. Basis Dis..

[11] Saygin C., Matei D., Majeti R., Reizes O., Lathia J.D. (2019). Targeting Cancer Stemness in the Clinic: From Hype to Hope. Cell Stem Cell.

[12] Williams E.L., Acquaviva C., Amoroso A., Chevalier F., Coulter-Mackie M., Monico C.G., Giachino D., Owen T., Robbiano A., Salido E. (2009). Primary hyperoxaluria type 1: Update and additional mutation analysis of the AGXT gene. Hum. Mutat..

[13] Kjersem J.B., Thomsen M., Guren T., Hamfjord J., Carlsson G., Gustavsson B., Ikdahl T., Indrebø G., Pfeiffer P., Lingjærde O. (2016). AGXT and ERCC2 polymorphisms are associated with clinical outcome in metastatic colorectal cancer patients treated with 5-FU/oxaliplatin. Pharm. J..

[14] Catarata M.J., Lourenço M., Martins M.F., Frade J., Pêgo A., Cordeiro C.R., Medeiros R., Ribeiro R. (2021). Pharmacogenetics of advanced lung cancer: Predictive value of functional genetic polymorphism AGXT Pro11Leu in clinical outcome?. Pulmonology.

[15] Pranzini E., Pardella E., Paoli P., Fendt S.M., Taddei M.L. (2021). Metabolic Reprogramming in Anticancer Drug Resistance: A Focus on Amino Acids. Trends Cancer.

[16] Muthusamy T., Cordes T., Handzlik M.K., You L., Lim E.W., Gengatharan J., Pinto A.F.M., Badur M.G., Kolar M.J., Wallace M. (2020). Serine restriction alters sphingolipid diversity to constrain tumour growth. Nature.

[17] Chen B., Cha J.H., Yan M., Cao N., Ye P., Yan X., Yang W.H. (2021). ATXN7L3B promotes hepatocellular carcinoma stemness and is downregulated by metformin. Biochem. Biophys. Res. Commun..

[18] Khillan J.S. (2014). Vitamin A/retinol and maintenance of pluripotency of stem cells. Nutrients.

[19] Seol H.S., Lee S.E., Song J.S., Rhee J.K., Singh S.R., Chang S., Jang S.J. (2016). Complement proteins C7 and CFH control the stemness of liver cancer cells via LSF-1. Cancer Lett..

[20] Mishra L., Banker T., Murray J., Byers S., Thenappan A., He A.R., Shetty K., Johnson L., Reddy E.P. (2009). Liver stem cells and hepatocellular carcinoma. Hepatology.

[21] Piao L.S., Hur W., Kim T.K., Hong S.W., Kim S.W., Choi J.E., Sung P.S., Song M.J., Lee B.C., Hwang D. (2012). CD133+ liver cancer stem cells modulate radioresistance in human hepatocellular carcinoma. Cancer Lett..

[22] Zhu R., Gires O., Zhu L., Liu J., Li J., Yang H., Ju G., Huang J., Ge W., Chen Y. (2019). TSPAN8 promotes cancer cell stemness via activation of sonic Hedgehog signaling. Nat. Commun..

[23] Novak D., Hüser L., Elton J.J., Umansky V., Altevogt P., Utikal J. (2020). SOX2 in development and cancer biology. Semin. Cancer Biol..

[24] Fregni G., Quinodoz M., Möller E., Vuille J., Galland S., Fusco C., Martin P., Letovanec I., Provero P., Rivolta C. (2018). Reciprocal modulation of mesenchymal stem cells and tumor cells promotes lung cancer metastasis. EBioMedicine.

[25] Brandi J., Pozza D.E., Dando I., Biondani G., Robotti E., Jenkins R., Elliott V., Park K., Marengo E., Costello E. (2016). Secretome protein signature of human pancreatic cancer stem-like cells. J. Proteom..

[26] Al-Dhfyan A., Alhoshani A., Korashy H.M. (2017). Aryl hydrocarbon receptor/cytochrome P450 1A1 pathway mediates breast cancer stem cells expansion through PTEN inhibition and β-Catenin and Akt activation. Mol. Cancer.

[27] Matsuda Y., Miura K., Yamane J., Shima H., Fujibuchi W., Ishida K., Fujishima F., Ohnuma S., Sasaki H., Nagao M. (2016). SERPINI1 regulates epithelial-mesenchymal transition in an orthotopic implantation model of colorectal cancer. Cancer Sci..

[28] Sun Y., Li W., Shen S., Yang X., Lu B., Zhang X., Lu P., Shen Y., Ji J. (2019). Loss of alanine-glyoxylate and serine-pyruvate aminotransferase expression accelerated the progression of hepatocellular carcinoma and predicted poor prognosis. J. Transl. Med..

[29] Chen P., Wang F., Feng J., Zhou R., Chang Y., Liu J., Zhao Q. (2017). Co-expression network analysis identified six hub genes in association with metastasis risk and prognosis in hepatocellular carcinoma. Oncotarget.

[30] Peiris-Pagès M., Martinez-Outschoorn U.E., Pestell R.G., Sotgia F., Lisanti M.P. (2016). Cancer stem cell metabolism. Breast Cancer Res..

[31] Baksh S.C., Todorova P.K., Gur-Cohen S., Hurwitz B., Ge Y., Novak J.S.S., Tierney M.T., Dela Cruz-Racelis J., Fuchs E., Finley L.W.S. (2020). Extracellular serine controls epidermal stem cell fate and tumour initiation. Nat. Cell Biol..

